# Experimental evidence characterizing pressure fluctuations at the seafloor-water interface induced by an earthquake

**DOI:** 10.1038/s41598-018-34578-2

**Published:** 2018-11-06

**Authors:** Hiroyuki Matsumoto, Toshinori Kimura, Shuhei Nishida, Yuya Machida, Eiichiro Araki

**Affiliations:** Research and Development (R&D) Center for Earthquake and Tsunami, Japan Agency for Marine-Earth Science and Technology (JAMSTEC), 2-15, Natsushima, Yokosuka 237-0061 Japan

## Abstract

An unusual combination of a laboratory experiment and *in situ* measurement of pressure fluctuations during an earthquake allows us to resolve some uncertainties in bottom pressure recorders (BPRs). *In situ* BPRs are usually contaminated by seismic waves during earthquakes; thus uncertainty still remains in the data obtained from BPRs. We examine *in situ* BPR data together with pressure variations produced by a dead weight (a pressure standard) in a laboratory experiment during an earthquake. The features recorded by the *in situ* BPRs are analysed as part of the overall experiment. We demonstrated that a 10-kg dead weight on a piston-cylinder across an area of 10 mm^2^ is capable of reproducing pressure fluctuations at a depth of 1000 m in the water column. The experiment also indicates that the internal mechanics of BPRs are isolated from incident seismic waves, suggesting that BPRs measure true *in situ* pressures without instrumentally induced disturbances. This constitutes the first instance in which pressure fluctuations recorded by *in situ* BPRs during an earthquake were reproduced using a pressure standard in the laboratory.

## Introduction

Bottom pressure recorders (BPRs) are essential instruments in geophysical and natural hazard applications such as the detection of tsunamis^1–3^, the observation of crustal deformation due to earthquakes^4–6^ or pre-earthquake movements^7^, and the monitoring of pore fluid pressure changes related to slow-slip seismic events^8,9^. Commonly utilized BPRs are divided into two types according to their internal mechanics: the Bourdon tube-type BPR^10^ and the thickness-shear mode resonator (TSMR) BPR^11^. This study is focused on Bourdon tube-type quartz pressure transducer BPRs, which record pressure changes by measuring the oscillations of a quartz crystal attached to the tip of a Bourdon tube by compensating for thermal effects. Two sets of quartz crystal frequencies associated with the temperature and pressure are transformed into physical pressure data based on a pre-calibrated coefficient. This method allows us to obtain highly precise pressure measurements under statically changing ambient pressures^12^ and contribute to scientific discoveries or new insights in hydrothermally active areas^13–15^. However, some uncertainties still remain in the output of a BPR during an earthquake; as a consequence, whether the BPR data acquired during an earthquake originated from true *in situ* ambient pressure changes or from the seismic response of an internal elastic element cannot be determined. The reason for this is twofold: first, we cannot monitor how an *in situ* BPR responds to an earthquake; second, it is difficult to determine how the balance weight attached to the Bourdon tube for reducing the orientation sensitivity affects the oscillations of a quartz crystal. Furthermore, detailed discussions regarding the output of a BPR during an earthquake cannot be found in the literature with the exception of a few previous studies^16–20^.

**Figure 1:**
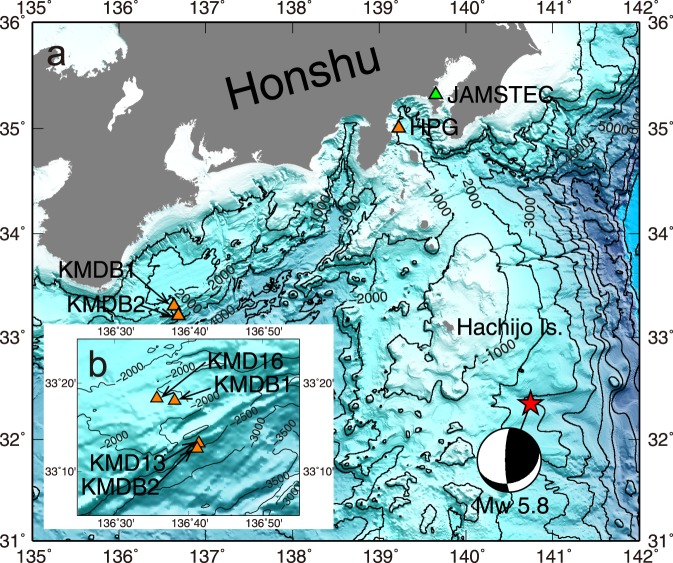
Map showing locations of the earthquake source and the *in situ* observatories. (**a**) The earthquake occurred off of Hachijo Island with a moment magnitude of 5.8, the epicentre of which is indicated by a red star. Orange triangles represent the *in situ* observatories; KMDB1 and KMDB2 are the long-term borehole monitoring system (LTBMS) observatories in which the bottom pressure recorders (BPRs) and the broadband seismometers are deployed, while HPG is the *in situ* BPR near JAMSTEC. Green triangle represents the laboratory where the experiment was conducted. (**b**) Special attention is paid to KMD16 and KMD13, which are the nearest DONET seafloor observatories to the *in situ* BPRs of KMDB1 and KMDB2, respectively. This map was created with the Generic Mapping Tools (GMT) software^37^.

Prior to the occurrence of a moderately strong earthquake to the southeast of Hachijo Island at 09:43 UTC on 16 November 2017 (Fig. 1), our team evaluated the long-term stabilities of various types of *in situ* sensors prior to their deployment in either the deep sea or a borehole. The earthquake was assigned a moment magnitude of 5.8 by the Japan Meteorological Agency (JMA), and thus, it was not a highly significant event. However, we were able to obtain an experimental dataset at the laboratory approximately 350 km away. This dataset consisted of three BPRs with an applied hydrostatic pressure of 10 MPa, three additional BPRs in a barometric pressure environment, and one broadband seismometer. These experimental observations provided physical clues for resolving the recorded response of an *in situ* BPR during an earthquake.

Therefore, in this study, we examine the BPR data recorded during the abovementioned earthquake in terms of *in situ* and experimental observations. Regarding the former, we analyse the pressure and seismic data from the long-term borehole monitoring system (LTBMS)^21,22^ and we compare those data with experimental observations conducted during the same earthquake. This is the first opportunity for which underwater ambient pressure data from both *in situ* observations and experimental observations are simultaneously available, and this unique opportunity affords us the ability to resolve measurement uncertainties regarding *in situ* BPR records.

## Results

### *In situ* BPR observations

**Figure 2:**
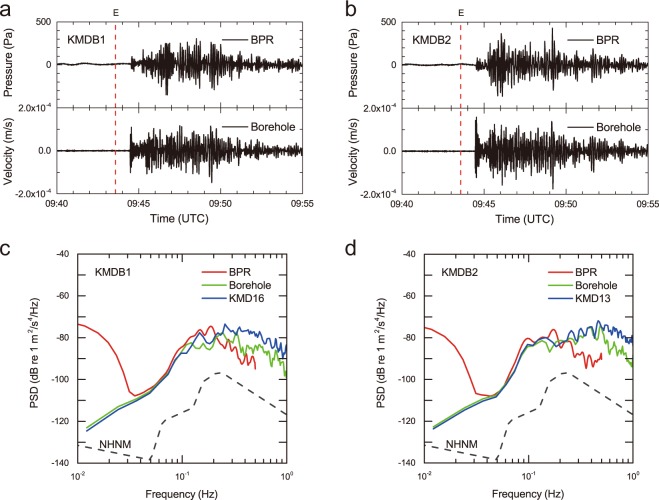
*In situ* records obtained from the LTBMS BPRs and their analysis. (**a**) Pressure and seismic waveforms recorded by LTDMS during the earthquake at KMDB1. Dashed line labelled by E indicates the earthquake origin. (**b**) Pressure and seismic waveforms of KMDB2 in the same form as (**a**). (**c**) Power spectral densities (PSDs) of the BPR, borehole broadband seismometer, and seafloor broadband seismometer denoted by BPR, Borehole, and KMD16, respectively, from KMDB1. Dashed line represents a new high noise model^38^. (**d**) PSDs of KMDB2 in the same form as (**c**). Analysis suggests that the BPRs follow the seafloor acceleration in the intermediate frequency range.

The LTBMS incorporates a multilevel pore fluid pressure sensing unit, a volumetric strain-meter, a tilt-meter, a broadband seismometer, three-component accelerometers, and a thermistor array, for the purpose of precise geophysical and geodetic observations below the seafloor, in which the BPR is included as a pressure sensing unit. Two LTBMS observatories indicated by KMDB1 and KMDB2 in Fig. 1 were able to capture all of the signals originating from the earthquake. The water depths of KMDB1 and KMDB2 are 1966 m and 2523 m, respectively. The BPRs addressed in this study are deployed at the top of the borehole, i.e., on the seafloor and always measure the ambient pressure of the seafloor, while the broadband seismometers are deployed at 907 m and 571 m deep below the seafloor at KMDB1 and KMDB2, respectively. The depths at which the borehole sensors are installed vary between two LTBMS observatories, but their *in situ* observation instruments are identical. The sampling frequencies of the *in situ* observations by the BPRs and the LTBMS seismometers are 1 Hz and 200 Hz, respectively. The pressure and seismic waveforms acquired via the LTBMS during the earthquake and their analysis are shown in Fig. 2. From the point of view of the BPRs data, apparent tsunami signals cannot be recognized (Supplementary Figs 1 and 2), therefore this is classified as not being a tsunamigenic earthquake.

The BPR and its below borehole broadband seismometer waveforms are plotted in Fig. 2a,b, respectively, in which we remove the tide component from the BPR waveforms by assuming a linear trend during the seismic arrivals. The seismometer components shown are the vertical ones. The epicentral distances to the LTBMS observatories range between 460 km and 470 km. The first seismic wave arrivals can be recognized at both LTBMS observatories between 09:44 UTC and 09:45 UTC. It is evident that the pressure fluctuations are observed simultaneously with the seismic wave arrivals. One of the objectives of this study is to prove whether BPRs can measure the true pressure fluctuations induced by seafloor oscillations. Hence, we compare the power spectral densities (PSDs) of the pressure waveforms with the seismic waveforms obtained at the nearby location. The BPR dataset and the seismometer recordings from 09:44 UTC to 09:49 UTC are used for this analysis. Assuming a quasi-flat seafloor, the vertical component of seafloor motion (namely the vertical acceleration) is related to the pressure fluctuation in the water layer. In this study, we consider only the vertical components of the borehole broadband seismometers. Relevant borehole seismic waveforms and their spectrograms are shown in Supplementary Fig. 3. Additionally, nearby seafloor seismic observation data from two observatories, KMD16 and KMD13 are selected from the DONET seafloor observatory^23^ since the BPR measures the ambient pressure of the seafloor. KMD16 is located 5 km west from KMDB1 and deployed at a water depth of 1970 m, while KMD13 is located 1 km northeast from KMDB2 and deployed at a water depth of 2441 m (Fig. 1b). The epicentral distances of KMD16 and KMD13 can thus be regarded as almost the same as those of KMDB1 and KMDB2, respectively, but the installation environment is quite different, particularly the depth. Each DONET observatory comprises two types of seismometers, i.e., a broadband seismometer and a seismic accelerometer. The former of the two seismometers is primarily used to detect slow-slip events or low-frequency seismic activity^24^, whereas the latter one is used to record strong ground motion^25^. The sampling frequency of the DONET seismometers is 100 Hz. Here we employ the broadband seismometer which has the same specification in terms of the sensitivity response as the seismometer of the LTBMS’s. The PSDs of the BPRs, the borehole broadband seismometers at KMDB1 and KMDB2, and the nearby DONET seafloor broadband seismometers at KMD16 and KMD13 are compared in Fig. 2c,d, respectively, in which the PSDs of the three instruments are indicated as BPR, Borehole, and accordingly either KMD16 or KMD13, respectively.

The PSDs of the two seismic sensors, i.e., the borehole and the seafloor broadband seismometers, perfectly coincide with each other in the frequency range below 0.1 Hz. The PSDs of the DONET seismic sensors are approximately 10 dB higher than those of the LTBMS seismic sensors in the frequency range above 0.3 Hz and 0.5 Hz for KMDB1 and KMDB2, respectively. The reason for the PSDs exhibiting a 10 dB difference between DONET and the LTBMS is that the sediment layer amplifies the seismic signals in the above mentioned frequency range. These observations prove that the deeper seismic sensor measures seismic waves of lower amplitude. The PSDs also show the similarity between the seafloor and the borehole seismic observations at the same locations. The installation depth of the LTBMS seismic sensor of KMDB1 (i.e., 907 m deep below the seafloor) is much deeper than that of KMDB2 (i.e., 571 m deep below the seafloor). The young accreted sediment layer (i.e., a relatively low velocity structure zone) of KMDB1 is thicker than that of KMDB2^26^, consequently frequency fluctuations dependent on the site layer thickness are observed in a broader frequency range. However, as such seismic observations are beyond the scope of this study, a detailed discussion of these observations is deferred for presentation elsewhere.

The most prominent feature of the PSDs revealed by comparing the PSDs of the BPRs with those of the seismic sensors (borehole and DONET) is that the BPR PSDs follow those of the seismometers in the intermediate frequency range above 0.04 Hz and gradually increase below 0.04 Hz relative to those of the seismometers. This is because BPRs also measure infragravity waves^27^, which have a predominant frequency that is lower than 0.05 Hz^28^. Since the BPRs are deployed on the seafloor, i.e., on the sediment layer, it is appropriate to compare with the seafloor seismometer rather than the borehole’s seismometer to clarify the incident seismic signals to the BPR. We should otherwise compare with the borehole seismometer taking into account the effect of sediment layer amplification. The correlation between the PSDs of the BPRs and seafloor (DONET) seismometers can be recognized up to approximately 0.2 Hz. This upper frequency limit varies as a result of the fundamental frequency of hydroacoustic resonances, which depends on the water depth according to previous studies^17,19^. The fundamental frequency *f* is provided theoretically as follows:

1 $${f}=\frac{{c}}{4{H}}$$

﻿where *c* and *H* are the sound velocity in the water column and the water depth, respectively. Equation (1) shows that the fundamental frequency of hydroacousitc resonances decreases with increasing water depth. Assuming an ideally rigid (i.e., decoupled) boundary between the seafloor and the water layer, this theoretical fundamental frequency is 0.19 Hz and 0.15 Hz for KMDB1 and KMDB2 (or accordingly KMD16 and KMD13), respectively, which corresponds to the *in situ* observations. The pressure should follow the seafloor velocity in the range of frequencies higher than the fundamental frequency of hydroacoustic resonance because hydroacoustic waves can travel through the water layer. Thus, we can demonstrate that the BPR pressure signals captured during the earthquake can be explained by induced vertical seafloor acceleration. However, we are not able to address the presence of instrumentally induced disturbances within the BPR, which should be isolated from the seafloor and thus insensitive to motion during an earthquake, within the *in situ* observations.

### Experimentally reproduced pressure fluctuations

**Figure 3:**
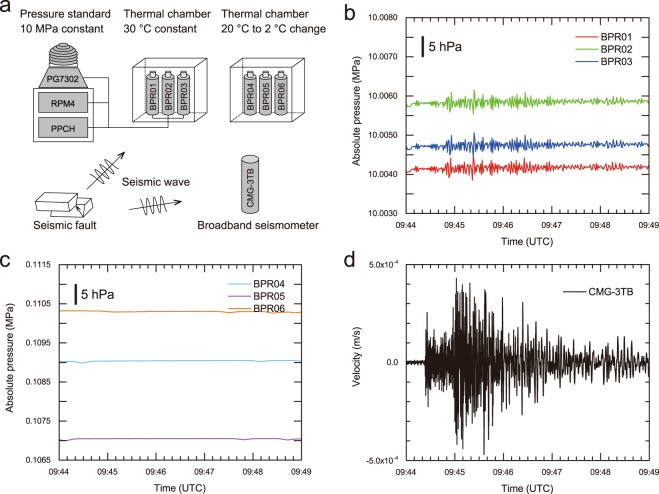
Experimental setup and the data acquired during the earthquake. (**a**) Pressure standard-reproduced hydrostatic pressure via the dead weight mounted on the piston-cylinder module during the earthquake. Three BPRs subjected to a hydrostatic pressure of 10 MPa were examined in a thermal chamber with a constant temperature of 30 °C; three other BPRs were examined in a barometric environment with a gradual thermal change from 20 °C to 2 °C. Broadband seismometer data were also examined simultaneously. (**b**) Waveforms obtained from the pressurized BPRs. Because the BPRs are absolute pressure transducers, the offset between adjacent BPRs denotes the instrumental tolerance. (**c**) Waveforms obtained from the unpressurized BPRs. Each BPR measures the barometric pressure change in the present condition. (**d**) Seismic waveform on the vertical component obtained from the broadband seismometer. Time stamps of the BPRs are determined using a computer, while those of the broadband seismometer are determined using a GPS clock.

The earthquake occurred while the long-term stabilities of six BPRs were being examined in a laboratory equipment. The experiment was conducted at the Japan Agency for Marine-Earth Science and Technology (JAMSTEC) location in Fig. 1a. For the long-term stability assessment, we examined the pressure sensing modules of the BPRs (i.e., we used the internal pressure sensing units prior to being manufactured as a BPR). However, these pressure sensing modules show the same specifications as the *in situ* LTBMS BPRs except for their repeatability and hysteresis; hence, we also refer to this module as a BPR, even though the instrument has not yet been manufactured in the laboratory. A precise hydrostatic pressure of 10 MPa (corresponding to a water depth of 1000 m) produced by the dead weight (i.e., the pressure standard) was applied to three of the BPRs via their pressure ports in a thermally controlled chamber with a constant 30 °C temperature; meanwhile, the other three BPRs were placed in different thermally controlled chambers and inspected to investigate thermal effects on BPRs in a barometric environment. The first three and last three BPRs are labelled BPR01, BPR02, BPR03 and BPR04, BPR05, and BPR06, respectively, in order to distinguish the BPRs according to their different environments. The setup of the key experimental equipment is summarized in Fig. 3a. This configuration of the BPR experiment subjects BPR01 through BPR03 to a pressure of 10 MPa plus the barometric pressure, while BPR04 through BPR06 are subjected only to the barometric pressure. The output of the BPRs followed the change in the barometric pressure before the earthquake. During the earthquake, high-frequency pressure fluctuations appeared in the records from BPR01 through BPR03 (Fig. 3b). However, BPR04 through BPR06 did not record such fluctuations (Fig. 3c), indicating that fewer effects of the earthquake were registered in their data. It is experienced that BPRs are affected by the gravity of the Earth as the output varies depending on the attitude of the sensor against rotation for its longitudinal-axis^29^. The attitude of all six BPRs examined in the laboratory was set to be aligned with the vertical direction for their longitudinal-axis. This orientation was also maintained during the earthquake. For this reason, it was possible to neglect the effect of attitude in the experiment. An initial slight drop in the pressure attributable to sensitive effects of the re-acceleration of the dead weight can be identified at 09:44 UTC for the pressurized BPRs. The thermal conditions for BPR04 through BPR06 became gradually colder (from 20 °C to 2 °C) during the earthquake. However, this gradual thermal change is negligible since the thermal effect on the BPR output can be compensated in this study. Note that the BPR time stamp is determined via a computer clock because a precise time was not required for the assessment of the long-term instrumental stability.

A borehole broadband seismometer manufactured by Güralp Systems Ltd. (model CMG-3TB) was also being examined during the earthquake (Fig. 3a). The sampling frequency of the broadband seismometer in the laboratory experiment is 100 Hz. The waveform recorded on the vertical component of the broadband seismometer is plotted in Fig. 3d. The time stamp for the broadband seismometer was determined using a GPS clock. Thus, the time difference (which is less than a few tens of seconds) between the BPRs and the broadband seismometer is shown. However, we do not correct for this time difference in this study because the most important goal here is not to determine each seismic phase arrival but to analyse the physical principle responsible for the pressure fluctuations in the BPR data.

A dataset from the K-NET strong-motion seismograph network in Japan^30–32^ was also partially available to validate the dataset obtained from the broadband seismometer. The nearby K-NET stations are displayed in Supplementary Fig. 4, and the available waveforms are plotted in Supplementary Fig. 5a. Although the K-NET dataset is only partly available, the PSD of the CMG-3TB broadband seismometer is easily comparable to those of nearby K-NET stations in the entire frequency range (Supplementary Fig. 5b). Thus, the K-NET data suggest that the JAMSTEC broadband seismometer was able to effectively capture the ground motions during the earthquake, providing evidence that seismic-induced ground motion indeed manifested itself within the laboratory. The experiment in which the unpressurized BPRs (i.e., BPR04 through BPR06) were examined simultaneously proved that neither the Bourdon tubes nor the quartz crystals in the BPRs were shaken by external forces due to the seismicity.

## Discussion

### Manifestation of pressure fluctuations

**Figure 4:**
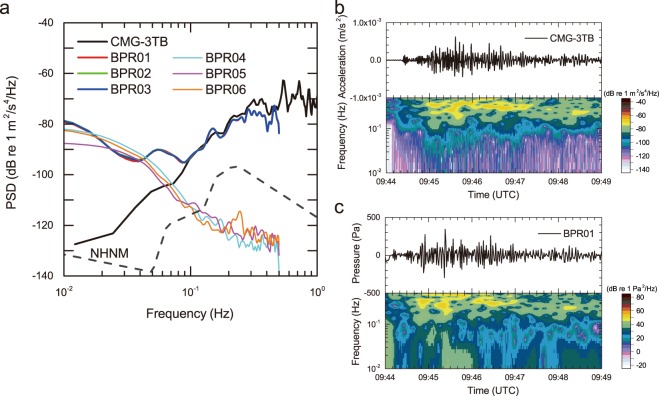
PSDs and spectrograms processed from the acquired experimental data. (**a**) Comparison of the PSDs from the pressurized BPRs, unpressurized BPRs, and broadband seismometer. Pressurized BPR PSDs follow that of the broadband seismometer in the high frequency range above 0.1 Hz, while those of the unpressurized BPRs are isolated from the seismic wave. (**b**) Spectrogram of the broadband seismometer converted to units of acceleration processed using a low-pass filter with a cut-off frequency of 0.5 Hz. (**c**) Spectrogram of BPR01, one of the pressurized BPRs. For the processing of BPR01, the barometric pressure contribution is removed by assuming a linear trend during the seismic arrivals. Based on a comparison of the spectrograms between the broadband seismometer and BPR01, the intensity patterns are similar in the high frequency range above 0.1 Hz.

The pressure fluctuations were recorded only by the pressurized BPRs via the pressure standard, even though the unpressurized BPRs were located within the same laboratory and received the same seismic wave. The hydrostatic pressure applied to the BPRs was produced by a 10-kg dead weight mounted on a piston-cylinder across an area of 10 mm^2^. We compare the PSDs of the three pressurized BPRs, the three unpressurized BPRs, and the broadband seismometer in units of acceleration (Fig. 4a). Since the sampling frequency of the BPRs is 1 Hz, we can calculate PSDs up to the Nyquist frequency of 0.5 Hz. These PSDs suggest that the three pressurized BPRs (i.e., BPR01, BPR02, and BPR03) are virtually indistinguishable, indicating that the same pressure fluctuations were recorded during the earthquake. The PSDs of the pressurized BPRs increase in the high frequency range above 0.1 Hz, whereas those of the unpressurized BPRs decrease with increasing frequency. The variations in the PSDs of BPR04, BPR05, and BPR06 are within a few dB and may be attributed to the ambient thermal effect caused by the experiment during the earthquake. Furthermore, the PSDs of all of the BPRs increase, although less markedly, in the low frequency range below 0.1 Hz due to the superposition of changes in the long-period barometric pressure onto the acquired pressure dataset.

Based on a comparison of the BPR PSDs with that of the broadband seismometer, it is evident that the PSDs of the pressurized BPRs follow the broadband seismometer PSD at frequencies above 0.1 Hz, albeit with a relatively narrow frequency band (i.e., with regard to the *in situ* observations) since there was less coupling in the BPRs compared to the broadband seismometer in the low frequency range below 0.1 Hz during the experiment. The BPRs addressed in this study are absolute pressure sensors, hence they always record ambient pressure change. Comparison of PSDs between the *in situ* observation and the experiment suggest that the water column of a few km deep contributes to the extension of the coincidence frequency range down to 0.4 Hz (Fig. 2b,c). In contrast, the PSDs of the unpressurized BPRs are not coincident with the PSD of the broadband seismometer.

### Contributions from seismic waves

Variations in the incident seismic waves and their similarities to the pressure waveforms are examined. The waveforms and their spectrograms from the broadband seismometer and from BPR01 are processed in Fig. 4b,c, respectively. Because the fluid pressure fluctuation is associated with the acceleration, the original broadband seismometer data are converted into units of acceleration; in addition, since the final acquired BPR dataset is sampled at a frequency of 1 Hz, a low-pass filter with a cut-off frequency of 0.5 Hz is also applied to the original seismometer dataset. Thus, the processed waveforms from the broadband seismometer and the associated spectrogram are plotted in Fig. 4b. For the processing of BPR01, meanwhile, we remove the barometric pressure contribution by assuming a linear trend during the seismic arrivals (Fig. 4c). This processing allows us to clarify the contributions from the incident seismic waves since the barometric pressure contribution to the BPRs is relatively large. Based upon a comparison of the two spectrograms, the intensity patterns of the major signals above 0.1 Hz from BPR01 correspond well with those from the broadband seismometer. Pressure fluctuations were also recognized by the other two pressurized BPRs in the low frequency range below 0.1 Hz from 09:45 UTC to 09:46 UTC, whereas these signals were not recorded by the broadband seismometer. This might be due to human activity associated with the response to the earthquake in the laboratory, which might have affected the pressure standard.

### Validation of the experiment

As discussed above, the present experiment appears to reproduce the pressure fluctuations recorded by the *in situ* BPRs during the earthquake. The experiment can easily reproduce the *in situ* hydrostatic pressures because a dead weight on the piston-cylinder module is always accelerated by the constant gravitational force of the Earth. Therefore, the most pertinent argument is whether the *in situ* pressure fluctuations caused by the arrivals of seismic waves can be reproduced by the experiment. The water depths of KMDB1 (1966 m) and KMDB2 (2523 m) correspond to twice and 2.5 times the applied pressure of 10 MPa in the experiment. Although frequency dependencies should be taken into account, based on a comparison of the analysis from the *in situ* measurements and the experiment (Figs 2c,d and 4a, respectively), the correlation between the acceleration and the pressure is relatively good in the intermediate frequency range. In the high frequency range (>0.2 Hz), the amplitude of the incident seismic wave at the *in situ* area is approximately 10 dB smaller than that in the experimental area because of the different epicentral distances. Moreover, the predominant frequencies are also different between the *in situ* and JAMSTEC sites mainly due to path effects (Fig. 2c,d and Supplementary Fig. 5b).

Finally, we examine the BPR of the cabled seafloor observatory deployed at a water depth of 1176 m (HPG in Fig. 1a and Supplementary Fig. 4)^33^, which is close to the applied pressure of 10 MPa in the experiment. Although the specifications of the BPR regarding the full scale of the operating depth are slightly different, the internal mechanical design is essentially identical. The only disadvantage associated with HPG is that the cabled seismic sensor at the same location was not properly operational during the earthquake. Consequently, only a paper chart is available during the earthquake. Nevertheless, we can directly compare the pressure waveforms from HPG and BPR01 during the earthquake without a time correction (Supplementary Fig. 6a). The long-term trend caused by the tide is recognizable in the data from HPG, while the barometric pressure contribution is slightly superposed onto the data from BPR01. According to Eq. (2) as described in the Methods section, a deeper water depth provides a larger pressure amplitude if the incident wave is assumed to be the same. The contribution from the difference in the pressure between the water depth at HPG (1176 m) and the applied experimental pressure (10 MPa) is expected to be approximately 20%. According to Supplementary Fig. 6a, the pressure waveforms virtually follow this expectation. The PSDs are compared in units of acceleration (Supplementary Fig. 6b), which are similarly normalized by the water depth. The PSDs suggest that the incident seismic waves at HPG and the JAMSTEC sites were similar. However, further discussion is not possible for the pressure waveforms because the locations (i.e., water depths) are different.

A dead weight of 10 kg can reproduce the pressure fluctuations at a water depth of 1000 m during the earthquake. The experimental evidence suggests that pressure fluctuations recorded by the pressurized BPRs are the responses to vibrations of the dead weight mounted on the piston-cylinder module, while those recorded by the *in situ* BPRs are the responses of the water mass accelerated by seafloor oscillations. The pressure standard was primarily designed for the calibration of BPRs, and the present experiment was conducted for such a purpose. The earthquake allowed us to reveal some uncertainties within the operation of the BPRs, and the experiment has proven that the internal BPR mechanics are isolated from incident seismic waves. This experimental evidence suggests that BPRs measure true *in situ* pressures without instrumentally induced disturbances. Accordingly, the features recorded by the *in situ* BPRs can be interpreted through the experiment. This constitutes the first instance in which pressure fluctuations recorded by *in situ* BPRs during an earthquake were reproduced using a pressure standard in the laboratory.

## Methods

### Bottom pressure recorders (BPRs)

All of the BPRs addressed in this study are Bourdon tube-type quartz pressure transducers manufactured by Paroscientific Inc. A pressure applied to the Bourdon tube generates an uncoiling force applied as tension on the quartz crystal. The temperature of the quartz crystal compensates for the thermal effect. More detailed descriptions of the sensing mechanism and the internal design are provided elsewhere^34,35^. The product models used in the *in situ* observatories and the experiment are different, but their basic mechanical structures are identical. A pressure transducer model 8B7000-2-005 is employed as the BPR in the LTBMS, while model 410K-184 is examined in the experiment. The 410K-184 pressure sensing module, demonstrating a better performance with regard to the repeatability and hysteresis, is employed in the 8B7000-2-005 pressure transducer. A model 8B2000-I BPR, which has a smaller specified pressure range (<2000 m), is employed at HPG. The temperature and pressure frequencies for the BPRs are processed either internally or externally, the final outputs for which are datasets of 1 Hz for the LTBMSs, the experiment, and HPG.

### BPR data processing

For the BPR data processing in this study, we convert the pressure values to units of acceleration. The ratio of the pressure to the vertical acceleration of the seafloor is provided theoretically as follows^36^:

2 $$\frac{p}{\alpha }\,=\,\frac{\rho }{k}\,\tanh \,kH$$

where *p* and *α* are the amplitudes of the bottom pressure and vertical acceleration, respectively, and *ρ* and *k* are the density of the water layer and the vertical wavenumber, respectively. The compressibility of the water layer does not play an important role in low-frequency seafloor oscillations, i.e., *kH* ≪ 1, and thus, Eq. (2) is approximated by *p* = *ρHα*. This means that changes in the seafloor pressure excited by low-frequency seismic waves are proportional to the seafloor acceleration. Therefore, we process the PSDs from the obtained BPR data using this relationship between the pressure and vertical acceleration of the seafloor.

### Pressure standard

A pressure standard composed of an oil-operated piston gauge with a dead weight and piston-cylinder module (model PG7302, DH Instruments, Inc.), an automated hydraulic pressure calibration/controller (PPCH, DH Instruments, Inc.), and a reference pressure monitor (RPM4, DH Instruments, Inc.) are used in the experiment. The BPRs are set into thermally controlled oil chambers, which are prepared to maintain a constant thermal environment (Fig. 3a). The principle of the pressure standard system is as follows. The pressure is defined by balancing it against a known force acting on a known cross-sectional area. The known area is defined by a vertically mounted piston which is free to rotate around its longitudinal axis inside a cylinder and translate vertically, whereas the known force is applied to the piston by loading it with a known mass (i.e., the dead weight) subjected to acceleration due to the gravitational force of the Earth.

If the dead weight situated on the piston-cylinder module is floated up and rotated within any tolerance stroke by an equivalent pressure produced by the PPCH pressure calibration/controller, the reference pressure is regarded as being calibrated. The PPCH instrument automatically adjusts the piston position within a stroke of ±5 mm. Two major disturbances to the produced pressure on the PG7302 piston gauge are considered possible while it is in proper operation. The first is an accelerated rotation of the dead weight when its speed becomes slow; the second is an adjustment by the PPCH of the position at which the dead weight is mounted on the piston when it reaches the allowable stroke limit. In this way, a highly accurate constant pressure can be produced without interruption. Because the barometric pressure sensitivity is 1 Pa per Pa of force applied to the piston gauge, the barometric pressure is simultaneously superposed onto the BPR dataset during the experiment.

### Experimental Setup and Data Processing

In the present experiment, a dead weight of 9.915459 kg situated on an effective area of 9.8 mm^2^ of the piston-cylinder module was used to reproduce the hydrostatic pressure of 10 MPa. A dead weight is always accelerated by the gravitational force of the Earth, resulting in an equivalent force. The pressure is therefore defined by the gravitational force of the dead weight (i.e., the mass accelerated by the gravity of the Earth) divided by the effective area of the piston-cylinder. The allowable stroke of the piston-cylinder is ±5 mm in the vertical direction, and thus, the dead weight can move only in the vertical direction within the allowed stroke range. We assume that the pressure fluctuations would be excited by the vibration of the dead weight.

The received force associated with the vibration of the dead weight can be derived by multiplying the mass with the received acceleration based on Newton’s law. At the same time, the equivalent force must be the product of multiplying the fluid pressure fluctuation with the effective area. Hence, the received acceleration can be related to the mass, the fluid pressure fluctuation, and the effective area. Both the mass and the effective area are initially defined parameters, while the fluid pressure fluctuation is an observed value. Consequently, the acceleration of the dead weight can be simply derived from the obtained pressure fluctuations observed in the BPRs. In addition, this processing allows us to compare the BPRs with the broadband seismometer using the same physical units (i.e., of acceleration).

## Electronic supplementary material

Supplementrary information
