# Supplementary material for: Experimental evidence characterizing pressure fluctuations at the seafloor-water interface induced by an earthquake
Source: Sci Rep. 2018 Nov 6;8:16406. doi: 10.1038/s41598-018-34578-2 (PMC6219582; doi:10.1038/s41598-018-34578-2)
Supplement: Supplementary file 1 — Supplementrary information [file 41598_2018_34578_MOESM1_ESM.pdf]

Supplementary information

Experimental evidence characterizing pressure fluctuations at the seafloor-water interface induced by an earthquake

Hiroyuki Matsumoto[1,*], Toshinori Kimura[1], Shuhei Nishida[1], Yuya Machida[1] & Eiichiro Araki[1]

[1] Research and Development (R&D) Center for Earthquake and Tsunami
Japan Agency for Marine-Earth Science and Technology (JAMSTEC)
2-15, Natsushima, Yokosuka 237-0061, Japan

[*] Corresponding author (email: hmatsumoto@jamstec.go.jp)

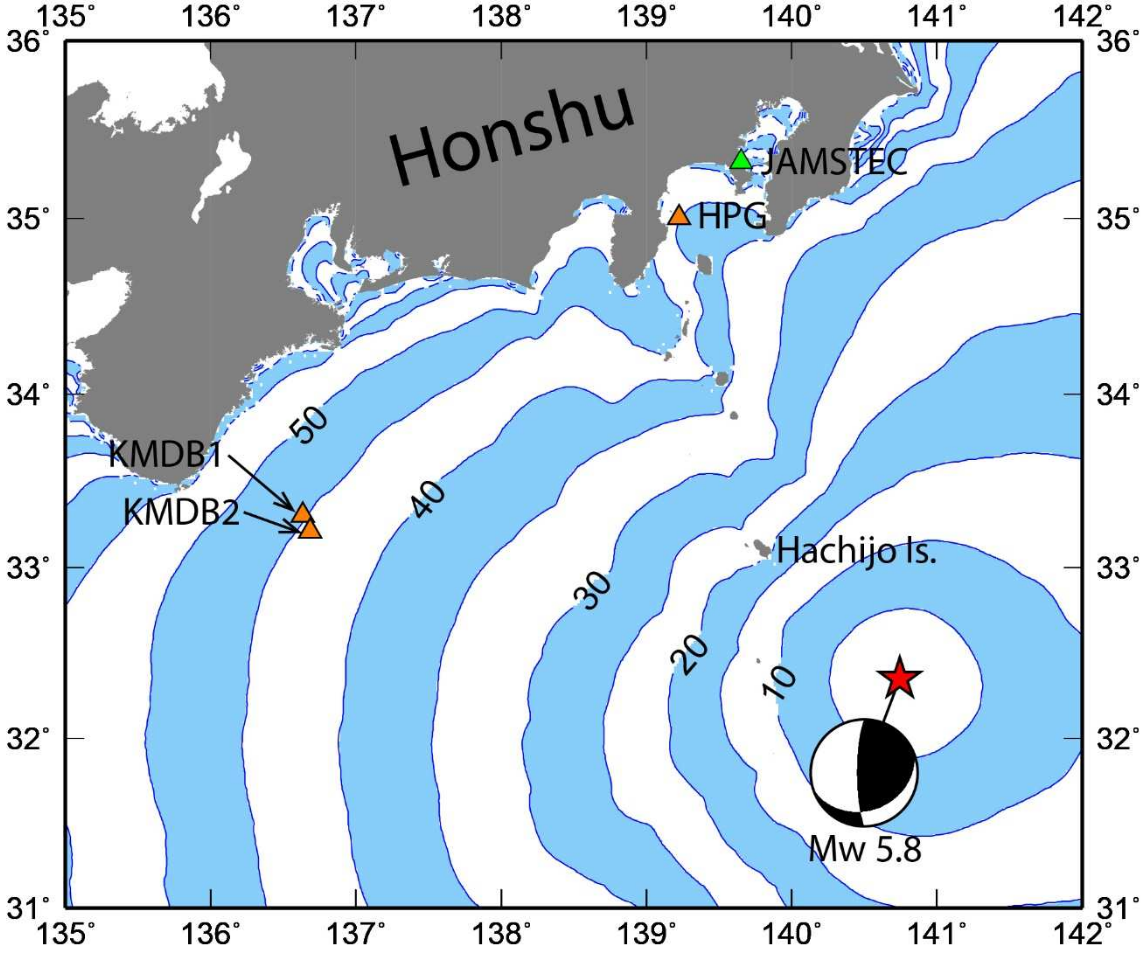

Supplementary Figure 1: Map showing the tsunami propagation patterns from the earthquake source, assuming that a tsunami was excited. Numbers superimposed on the stripe-pattern contour represent arrival times in minutes. Tsunami arrival times at long-term borehole monitoring system (LTBMS) observatories are 45 minutes after the earthquake. This map was created with the Tsunami Travel Time (TTT) software and the Generic Mapping Tools (GMT) software[37].

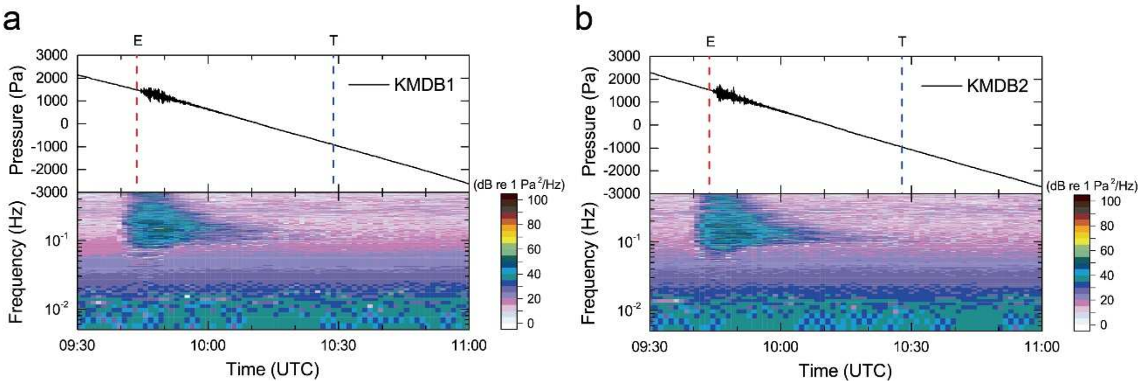

Supplementary Figure 2: Pressure waveforms and their spectrograms of (a) KMDB1 and (b) KMDB2. Vertical dashed lines labelled by E and T represent the earthquake origin and the expected tsunami arrival times, respectively. Pressure fluctuations induced by the seismic wave could be recorded, whereas no tsunami signals could be recognized at the expected arrival time. No other instrumentally recorded information supporting the presence of a tsunami was found elsewhere. This is why the present event was classified as not being a tsunamigenic earthquake.

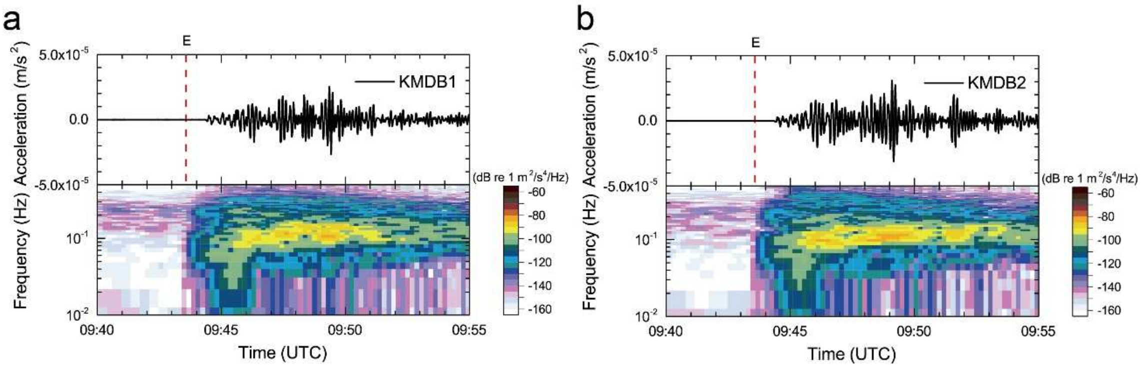

Supplementary Figure 3: Spectrograms of broadband seismometer of (a) KMDB1 and (b) KMDB2. Units are converted to acceleration, and a low-pass filter with a cut-off frequency of 0.1 Hz is applied to the acquired data. The vertical dashed line labelled by E represents the earthquake origin time. The low-frequency dispersive features can be seen from 09:45 UTC at the both observatories.

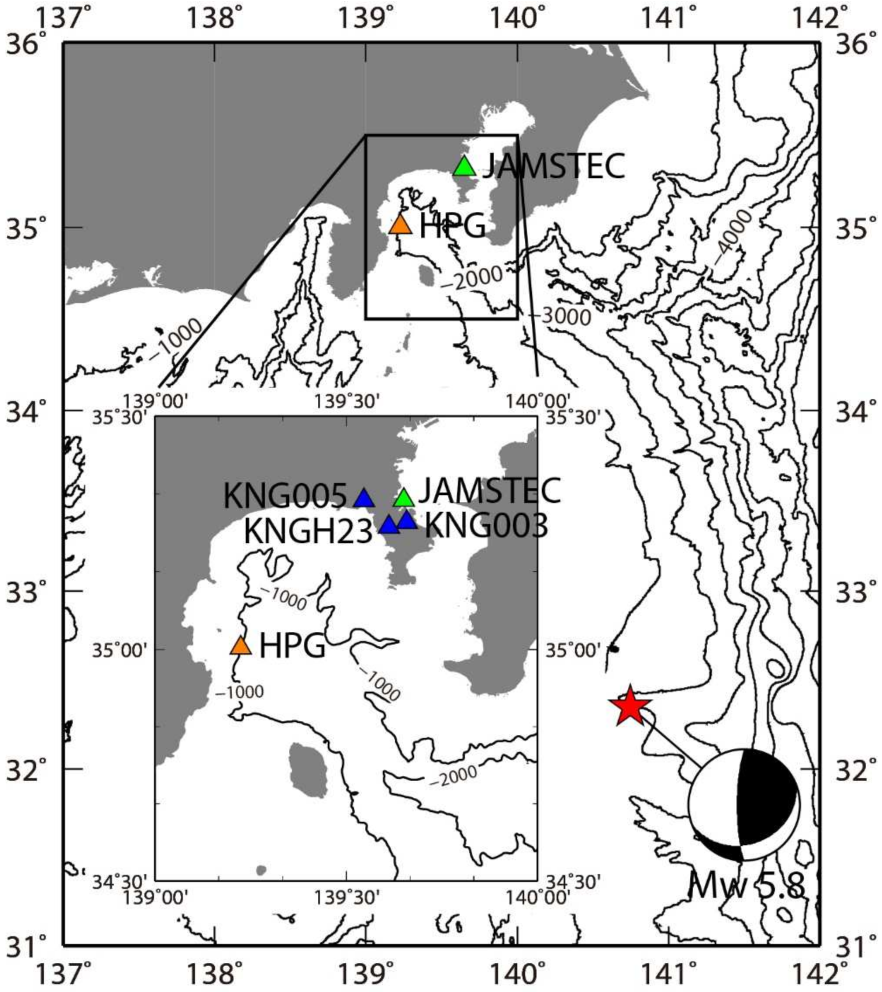

Supplementary Figure 4: Map showing the strong-motion seismograph network (K-NET) stations and the in situ BPRs near JAMSTEC denoted by the blue triangles and orange triangle, respectively. The green triangle shows the location of the experiment. This map was created with the Generic Mapping Tools (GMT) software[37].

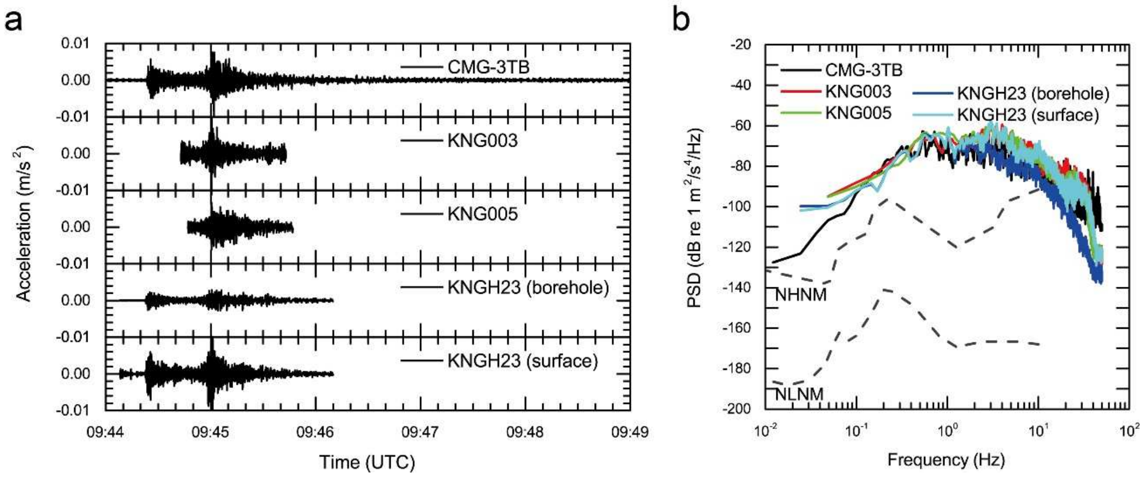

Supplementary Figure 5: Seismic waveforms from K-NET with a broadband seismometer and their PSDs. (a) Top panel shows the waveform obtained from the broadband seismometer, while the bottom four waveforms are obtained from K-NET. Ordinary K-NET stations are characterized by a seismic sensor deployed at the surface. KNGH23 has an ad hoc borehole sensor. K-NET data were available during this limited time period. (b) PSDs of seismic waveforms shown in (a). Dashed lines represent new high and low noise models[38]. These panels suggest that the broadband seismometers fully captured the complete seismic waveforms.

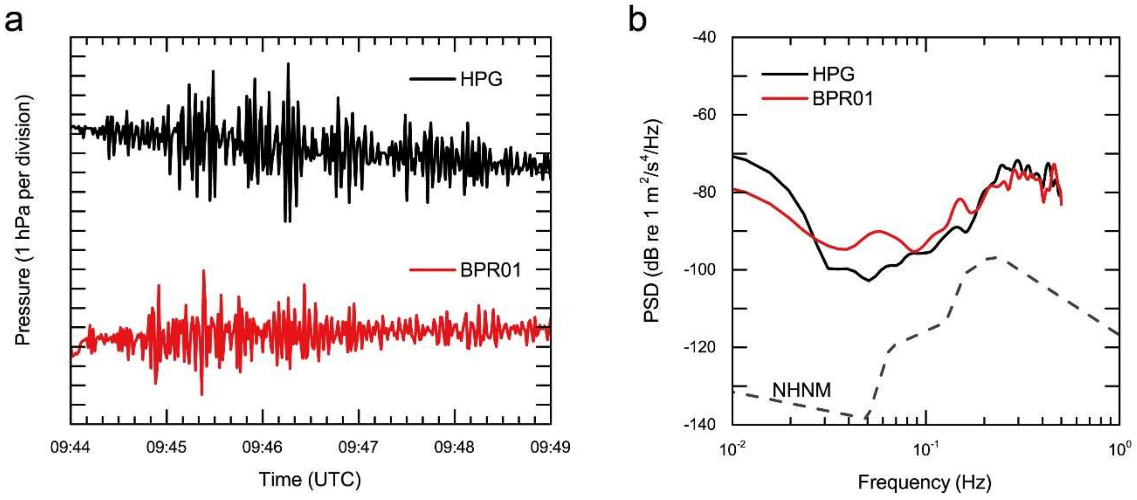

Supplementary Figure 6: Comparison of the in situ and the experimental observations. (a) Original pressure waveforms of HPG and BPR01, i.e., neither the tide component nor the barometric pressure were removed from the acquired data. (b) PSDs processed from the HPG and BPR01 waveforms. PSDs suggest that the incident seismic waves at HPG and BPR01 are similar.
